# The Medico-Psychological Association and Their Nurses

**Published:** 1897-02-13

**Authors:** 


### THE MEDICO-PSYCHOLOGICAL ASSOCIATION AND THEIR NURSES.

"M.D." writes: I have to ask you, in the exercise of your usual fairness, to give me a small space in which to offer a few comments on the voluminous contributions on this subject. Dr. White need not trouble himself regarding my acquaintance with, nor experience in, the home life of an asylum. This is outside the question altogether, and only tends to lay bare the poverty of Dr. White's argument. I have carefully read the remarks of your various correspondents, and it would seem that there is an idea on the part of hospital nurses and their supporters that the champions of asylum nurses desire to class them as equal in all respects with hospital nurses. Now, sir, if such an idea does exist, it can have no foundation, because not only is the training specialised, but the nature of their duties is altogether different to that of the hospital nurse. Let me strongly advise asylum nurses not to be led away by the unguarded statements of one or two faddists. Their claims and interests are in good hands. I do not mean those whose names are most frequently in print, but a large body of quiet and earnest workers who hate publicity, and detest notoriety. If I were an asylum nurse or attendant, I should feel strongly tempted to call aloud, "Save me from my friends." It is to Dr. Wood's credit that in his numerous letters he has used temperate and conciliatory language, which cannot be said for many of the other ponderous and professorial contributions. Dr. Robertson's lengthy letter reminded one of the hackneyed introductory lecture to first year students. It is through individual and isolated efforts that any permanent good can be effected in this matter, but Dr. Robertson's suggestions are neither new nor any advance. Statements which are hard to believe, and at variance with the opinion of ripe experience, would seem to be an occasional characteristic of communications from over the border. Perhaps Dr. Robertson has an electric machine for replacing ignorance and cocksureness with common sense and usefulness, for I can think of no other method or means by which the 5-minute transformation scene could be accomplished. We are informed that two years' training is the time required for the certificate of the Medico-Psychological Association. But surely if hospitals require three years' training before granting a certificate, it is equally necessary in the case of mental nurses. The phrase mental nurse is a most unfortunate one, because, while many might be capable of taking charge of a mental case in private practice, the majority are absolutely incapable of being made charge of a ward. There should be no attempt on the part of any authority to boycott or to hold up any system or any certificate as infallible. Any mental nurse who undergoes a thorough and complete system of training extending over a a period of three years, which is the time required in several institutions, is, in my humble opinion, a much better trained and more valuable woman than her hospital sister; but do not force her to join this or that society unless as a free act on her own part. Year by year a superior class of women are entering asylums as nurses, and as they are by far the stronger body of nurses in the country, I would venture to suggest, for the consideration of hospital nurses, that though their humbler sisters in the asylum world cannot talk of "My friend Lady X or Lady B," they are in every respect as fully competent, and, in their own special work, better qualified than the majority of hospital nurses. What, then, can be the objection to their registration as mental nurses? Another point, so far overlooked, is this: Will the registration of mental nurses be of any material advantage to the public asylums? I don't think so; but it will help the public who require private nurses, and for this reason alone the suggestion is worthy of all due consideration.

